# Urbanization influences the distribution, enrichment, and ecological health risk of heavy metals in croplands

**DOI:** 10.1038/s41598-022-07789-x

**Published:** 2022-03-09

**Authors:** Salar Rezapour, Sina Siavash Moghaddam, Amin Nouri, Kamal Khosravi Aqdam

**Affiliations:** 1 Soil Science Department, Urmia University, P.O. Box 165, 57134 Urmia, Islamic Republic of Iran; 2 Department of Plant Production and Genetics, Faculty of Agriculture, Urmia University, Urmia, Islamic Republic of Iran; 3 Hermiston Agricultural Research and Extension Center, Oregon State University, Hermiston, OR 97838 USA; 4 Department of Soil Science, Faculty of Agricultural Sciences, University of Guilan, Rasht, Islamic Republic of Iran

**Keywords:** Ecology, Plant sciences, Biogeochemistry, Environmental sciences, Solid Earth sciences, Risk factors

## Abstract

The contamination of urban soils with heavy elements due to the rapid development of urbanization and urban services has become a major environmental and human health challenge. This study provides insight into the urbanization controls on combined pollution severity and health risk potential of heavy metals in corn-cultivated urban versus non-urban soils. A multifaceted assessment was conducted using enrichment factor (EF), ecological risk (ER), bioconcentration factor (BCF), transmission factor (TF), hazard index (HI), and carcinogenic risk (CR). The results indicate a significant increase in the concentration of all metals in urban farmlands. When compared to the non-urban soils, EF implies a significant increase of all metals in the urban soil, downgrading this index from minimal enrichment (EF < 2) in the control soils to moderate enrichment (2 ≤ EF < 5) in the urban soils. Likewise, the average ER value showed an increase in the urban soils than in the control soils in the order of Fluvisols (66.6%) > Regosols (66.1%) > Cambisols (59.8%) > Calcisols (47%). The BCF and TF values for different elements decreased in the order of Cd (0.41–0.92) > Cu (0.1–0.23) > Zn (0.1–0.18) > Ni (0.01–0.03) > Pb (0.005–0.011) and Zn (0.75–0.94) > Cu (0.72–0.85) > Pb (0.09–0.63) > Cd (0.17–0.22) > Ni (0.01–0.21), respectively, which indicates that certain metals were not mobilized to the extent that they had been accumulated in the plant roots. The total carcinogenic risk was ranged from 5.88E−05 to 1.17E−04 for children and from 1.17E−04 to 2.30E−04 for adults, which implies a greater associated health risk for children.

## Introduction

Urban soils located within and around the suburban and marginal areas are key elements of urban ecosystems which play an essential role in the life quality of urban citizens. Green infrastructures provide multiple environmental services within urban settings, e.g., soil carbon storage, rainstorm buffering, and air quality improvement^1^. However, rapid urbanization and subsequent release of urban contaminants from anthropogenic activities, e.g., vehicle emissions, coal combustion, and waste disposal, imposes a progressive pressure on urban soil resources^2,3^. Many compounds and urban waste that find their ways into the soils contain element compounds, such as heavy metals known to be toxic and fatal to living organisms which may persist in urban soils for a long time, thereby threatening human, animal, and environmental health^4^. Even though urban soils constitute almost 3% of terrestrial land area, approximately 55% of the earth population as of 2018 reside in urban areas^1,5^.

Studies conducted around the world confirmed that heavy metal pollution in urban soils has been exacerbated in magnitude and extent over the last decades^2,4^. Cadmium (Cd) contamination in urban soils is reportedly severe in Asia and Australia^6^. The concentrations of copper (Cu), lead (Pb), and zinc (Zn) were within the pollution range in the urban soils of Mexico and Italy^7^. These metals in high concentrations in urban soils may damage crops directly by limiting photosynthesis, injuring roots, reducing growth, and ultimately, causing plant death^4^. Furthermore, the accumulation of heavy metals in these soils may impair soil quality, which may constrain the growth and yield of crops and trees in the affected regions^3^. On the other hand, these metals can get into the body of living organisms and humans by ingestion, inhalation, skin contact, and consumption of soil products, and initiate several diseases. Heavy metals cannot be metabolized inside the body of living organisms and may accumulate in various tissues—e.g., fats, muscles, bones, and joints, leading to a variety of diseases^8^. No contamination thresholds have been assigned for certain elements as they are harmful at any concentration. Heavy metals can accumulate in tissues and cause severe diseases in the long run. Pb and Cd are examples of these elements whose accumulation in human tissues can be extremely harmful to neural and enzymatic systems and skeletons and cause lung cancer and osteogenesis imperfecta^9,10^. Accordingly, the assessment of ecological risk and health risk of heavy metals in urban soils has emerged as a new challenge for researchers in most parts of the world. Heavy metals can enter the ecosystem of urban soils from different sources related to human activities. Some important examples include traffic emissions, coal and fuel combustion, industrial activities, metal melting facilities, plating, dying, mechanical activities, agricultural activities, sewage, industrial and domestic effluents, and so on^11,12^. The introduction of these compounds to urban soils not only increases the concentration of heavy metals but also brings synthetic compounds into these soils, complicating the behavior and properties of urban soils and their response to these elements^10^.

Urbanization has severely expanded in Iran over the recent decades. The urbanization is expected to further increase at a growing pace in the coming decades. The population of Iran has been quadrupled over the last 50 years, which has resulted in the significant expansion of urban areas, urbanization, the conversion of agricultural lands into residential areas, and the deployment of considerable urban waste. The industrial, service and trade facilities have simultaneously expanded in suburban areas. Accumulation and mobilization of urban waste pose a threat to human health, aquatic ecosystems, and urban habitats.

Previous works had showed that human and urbanization processes can result in the accumulation of heavy metals in water and soil near urban areas, but little is known about their effects through soil-crop-human health domain^2,3,6^. It is, therefore, essential to monitor and understand the fate, bioconcentration, translocation, and health risk of heavy metals in urban soils and ecosystems, which have been subject to fewer investigations than agricultural and natural soils. Corn is among the major grain crops grown in the vicinity of Iranian urban habitats. However, limited information is available regarding the pollution and health risk of heavy metals in the soil-crop-human domain in urban and sub-urban farmlands.

This study aimed to.

1. compare the concentration and accumulation of Zn, Cu, Cd, Pb, and Ni in the soil and corn grains between urban and non-urban areas,

2. estimate the concentration factor and translocation factor of heavy metals from the urban soil to the corn plants,

3. estimate metal exposures in adults and children through corn consumption in urban soils with different soil types, and

4. predict the carcinogenic and non-carcinogenic risk of heavy metals for local communities and determine whether this risk level requires remediation.

## Methodology

### Study site

**Figure 1:**
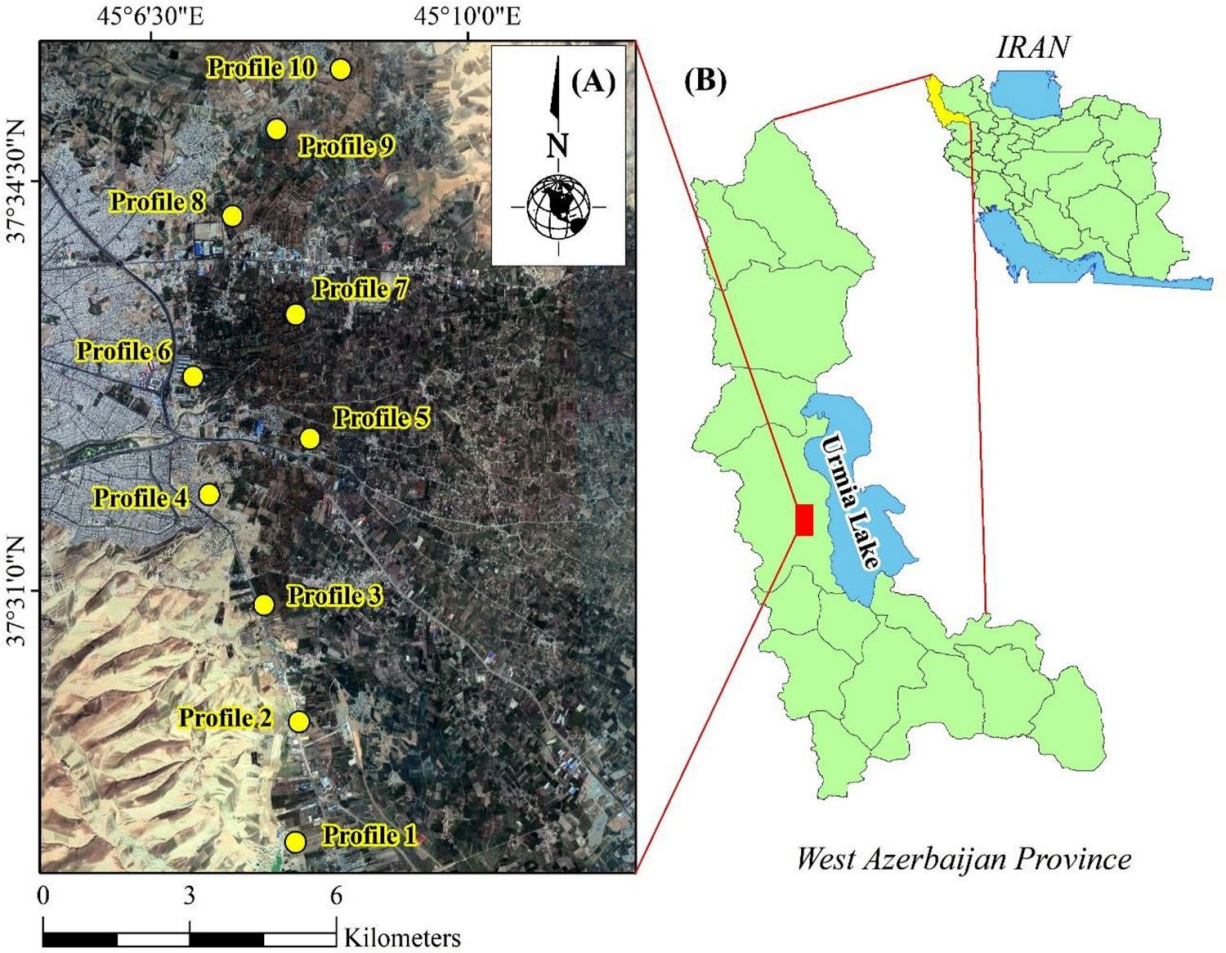
The location of the study area (https://www.qgis.org/ and https://www.google.com/earth/).

The study site is located in West Azerbaijan province in Iran (45° 07′ 20ʺ E to 45° 07′ 38ʺ E; 37° 31′ 44ʺ N to 37° 34′ 58ʺ N) (Fig. 1). According to the Köppen classification system, the region has a Mediterranean semi-arid climate with a Xeric moisture regime and a Mesic temperature regime. The main land use is agriculture and the dominant crops include grains, oilseeds, medicinal plants, vegetables, grapes, and apples. Irrigation is mostly performed by the flooding system. The surface waters leaving Urmia City and wells constitute irrigation sources. Overall, wheat and corn are the main grain crops, which are cultivated in rotation with other crops. This study was conducted on summer corn farms within the suburban areas over a growth period of about 120 days from June to September of 2019. The corn cultivar commonly cultivated in the region is single-cross 704 with a 5–8.5 t ha^−1^ approximate yield. This cultivar has been cultivated in the region for over five years. The agronomic operations include annual plowing, the application of organic and chemical fertilizers, and the application of chemical pesticides to control pests, diseases, and insects if required. The fertilization regime is composed of 40–50 t ha^−1^ rotten manure, 200–250 kg ha^−1^ urea, and 100–150 kg ha^−1^ triple superphosphate.

### Field campaign and soil sampling

In a field campaign, 10 soil profiles were allocated in the farmlands affected by urban activities. Soil profiles were excavated, and soil types and horizons were characterized within the soil profiles. According to the WRB classification system^13^, 4 major soil types including Calcisols, Cambisols, Fluvisols, and Regosols were identified across 10 soil profiles. The landscape characteristics (e.g., elevation, slope, drainage), as well as soil variations and the target crop, were the parameters considered in the selection of soil profiles and the sampling locations. Our primary goal was to include as much spatial variabilities as possible in the sampling scheme. Each soil profile was considered a central sampling location and four additional composite (across depth) soil samples were collected (0–0.5 m depth) at four cardinal directions each being 50–100 m from the central point (soil profile) (n = 50). Following the identical spatial considerations, 4 additional soil profiles were allocated, excavated, and characterized as control within the adjacent non-urban area. Each of 4 profiles corresponded to Calcisols, Cambisols, Fluvisols, and Regosols identified in the urban agroecosystem. An analogous soil sampling procedure was followed for the control soils as for the urban soils (n = 20). The explained procedure was based on a protocol designed and applied to multiple studies as a comprehensive regional project^10,14,15^. Clay and sand fractions ranged from 20 to 50% and from 10 to 50%, respectively, implying a wide range of soil textural classes from loam to clay in the study region. The soil samples were air-dried and passed through a 2-mm sieve before the analyses in the laboratory.

For each soil sampling site, five corn plant samples were randomly collected from both urban and non-urban soils at maturity stage. Following the separation of roots and grains, the samples were washed by distilled water, oven-dried at 65 °C for 48–72 h, ground with mortar and pestle, and then sieved through a 0.35-mesh sieve.

### Laboratory analyses

The distribution of soil particle sizes and texture was determined using a Bouyoucos hydrometer (Gee and Or, 2002). All soil samples were analyzed by^16^ standard procedure to measure pH, EC, and organic content. Soil exchange capacity was measured by the sodium acetate (NaOAc) method at pH 8.2^17^ and equivalent and active calcium carbonate by reaction with hydrochloric acid (Nelson, 1982) and ammonium oxalate^18^, respectively. Total N and available P were estimated by the Kjeldahl method^19^ and spectrophotometry^20^. The ion composition of the soil solution was determined in saturated extract^21^.

The total form of Zn, Cu, Cd, Pb, and Ni in the soil were extracted by nitric acid (Soon and Abboud, 1993). Also, to determine heavy metals in corn roots and grains, 1 g sample was ashed at 500 °C, extracted by 2-M hydrochloric acid^22^. The concentrations of the heavy metals in soil and corn plant extracts were measured by a Shimadzu 6300 atomic absorption spectrophotometer, which had been calibrated by the standard laboratory material. To assess the quality assurance (QA) and quality control (QC) of element analyses, reagent blanks, replicates, and standard reference materials (certified reference materials) was used. Their recovery was estimated at 94–103% for five heavy metals. To verify the accuracy of the measurements, the metal content was re-measured in 20% of the samples which their error was less than 5%. The detection limits were 2, 1, 0.01, 1, and 1 mg kg^−1^ for Zn, Cu, Cd, Pb, and Ni, respectively. Besides, the standard solution concentrations were 0.5, 1, 1.5, 2, and 2.5 or 4, 6, 8, 10, and 12 mg l^−1^ for Zn and Cu; and 0.5, 1,1.5, 2 and 2.5 mg l^−1^ for Cd, Pb, and Ni.

### Data analysis

Analysis of variance (ANOVA) was conducted using GLIMMIX procedure in SPSS 16^23^ and least squared means were compared using Duncan’s test at the P < 0.05 confidence level. A completely randomized design was used with 4 soil types for each of the urban and non-urban treatments (Table 1).

**Table 1:** Some environmental attibutes and soil classification of the current study.

Soil profile	Physiographic unit	Erosion	Slope class*	Drainage class*	Elevation (m)	Soil depth	Soil type
**Urban soil**							
1	RAP	E0	A	Well	1335	Very deep	Fluvisols
2	RAP	E1	A	Well	1307	Deep	Cambisols
3	RAP	E1	A	Well	1327	Moderatly deep	Regosols
4	RAP	E0	A	Well	1332	Very deep	Fluvisols
5	RAP	E1	A	Well	1306	Deep	Cambisols
6	RAP	E0	A	Well	1334	Very deep	Fluvisols
7	RAP	E1	A	Well	1326	Moderatly deep	Regosols
8	RAP	E0	B	Well	1314	Very deep	Calcisols
9	RAP	E0	B	Well	1314	Very deep	Calcisols
10	RAP	E0	B	Well	1314	Very deep	Calcisols
**Non- urban soil**							
1	RAP	E0	B	Well	1313	Very deep	Calcisols
2	RAP	E1	A	Well	1303	Deep	Cambisols
3	RAP	Eo	A	Well	1336	Very deep	Fluvisols
4	RAP	E1	A	Well	1325	Moderatly deep	Regosols

Moderatly deep, Deep, and Very deep: a depth of 50–80, 80–120, and more than 120 cm, respectively,

*Symbols applied based on Soil Survey Staff (2013).

### Soil pollution indices

The enrichment factor (EF) and ecological risk factor of the metals were calculated by Eqs. (1)–(3)^24,25^ to determine the range of pollution in the study soils.

1 $$\mathit{EF}= \frac{\left(\frac{{C}_{m}}{{C}_{s}}\right)x}{\left(\frac{{C}_{m}}{{C}_{s}}\right)C}$$

where *EF* is the enrichment factor, *C*_*m*_ is the heavy metal concentrations in soil (mg kg^−1^), *C*_*s*_ is the concentration of the terrestrial reference element at soil (mg kg^−1^), and C and X are heavy metal concentrations in the earth crust and soil, respectively^24,25^.

The enrichment factor was classified into minimal (EF < 2), moderate (2 ≤ EF < 5), significant (5 ≤ EF < 20), very high (20 ≤ EF < 40), and extremely high (EF > 40)^4,24,25^.

Pollution index and ecological risk factor were estimated by Eqs. (2) and (3), respectively.

2 $$PI=\frac{{C}_{m}}{{C}_{b}}$$

3 $$\begin{gathered} E_{i} = TiPI \hfill \\ E{\text{R}} = \sum E_{i} \hfill \\ \end{gathered}$$

where *PI* is the soil pollution index by Zn, Cu, Cd, Pb, Ni, *C*_*m*_ is heavy metal concentration in the soil, *C*_*b*_ is the reference value for metals, and E_i_ is the monomial potential ecological risk factor. *ER* is the ecological risk factor of metals, and *Ti* is the metal toxicity factor, which is equal to 5 for Cu, Pb, and Ni, 30 for Cd, and 1 for Zn^24,25^.

Also, the ER value was categorized as the low potential ecological risk for ER < 40, the moderate potential ecological risk for 40 ≤ ER < 80, the relatively high potential ecological risk for 80 ≤ ER < 160, high potential ecological risk for 160 ≤ ER < 320, and very high ecological risk for ER ≥ 320^24,25^.

### Bioconcentration and phytoranslocation factor in corn

The bioconcentration factor (BCF) and the phytotranslocation factor (TF), which are typically considered the most important indices to estimate the transferability of heavy metals from soil to crop, were calculated by Eqs. (4) and (5), respectively^4,10^.

4 $${\text{BCF }} = \, \ \frac{{{\text{C}}_{{{\text{root}}}} }}{{{\text{C}}_{{{\text{soil}}}} }}$$

5 $$TF=\frac{{C}_{grain}}{{C}_{root}}$$

where C_root_, C_grain_, and C_soil_ represent the measured concentration of heavy metals in the roots, grains, and soil of the corn plants, respectively.

### Assessment of heavy metals of health risk potential

The potential non-carcinogenic and carcinogenic health risks of heavy metals were estimated for local people by ingestion of corn grain in three age groups of children and adult men and women using Eqs. (6)–(8) as follows^26^:

6 $$DI_{{}} = C \times \frac{IR \times EF \times ED}{{BW \times AT}}$$

7 $$HQ=\frac{DI}{RfD}$$

where *DI* is the daily intake of heavy metals (mg kg^−1^ day^−1^); *C* represents the metal concentrations in corn grains (mg kg^−1^); *IR* represents ingestion rate (0.63 and 2.2 g day^−1^ for children and adults, respectively), estimated based on the value of corn consumed per meal; *EF* represents the exposure frequency (365 days' year^−1^); *ED* represents the exposure duration; *BW* represents the mean body weight; *AT* represents the average exposure time for non-carcinogenic effects (ED × 365 days' year^−1^); *RfD* represents reference oral dose for the metals^26^; and *HQ* represents the hazard quotient that estimates the non-cancer risk during a lifetime.

The sum of HQ amounts is expressed as Hazard Index (HI) representing the overall potential of non-carcinogenic effects^26^. The value of HQ or HI > 1 represents the adverse health impacts while there is the absence of adverse health impacts when their amounts are HQ or HI < 1.

Carcinogenic risk (CR) was estimated by Eqs. (8) and (9) to determine the likelihood of the emergence of a kind of cancer because of exposure to a carcinogenic metal^27^.

8 $${\text{CR}} = {\text{DI}} \times {\text{SF}}$$

9 $$TCR={\sum }_{i=1}^{n}CR$$

where CR represents the potential risk of an individual carcinogenic metal, SF is the slope factor (kg day mg^−1^), TCR represents the sum of cancer risk of heavy metals. The cancer risk is categorized as: no significant health risk (CR/TCR < 1E−06), admissible risk (1E−06 < CR/TCR < 1E−04), and inadmissible risk (CR/TCR > 1E−04).

## Results and discussion

### General characteristics of study soils

Table 2 presents the descriptive statistics regarding the soil characteristics. Significant changes were observed in the distribution of sand (110–850 g kg^−1^), silt (50–530 g kg^−1^), clay (100–610 g kg^−1^), and soil textural class (7 texture classes) showing the diversity of natural and human processes involved in the formation and development of these soils^28^. Almost all soil samples were alkaline (with reaction at a range of 7.4–8.1) and calcareous (with CCE at a range of 5.5–35%). The EC of some soils was > 4 dS/m (about 7% of the soil samples), indicating the partial salinity of the study soils. The organic carbon and total N contents of the soils were, on average, 2% (0.8–3.1%) and 0.28% (0.05–0.51%), respectively, placing them within the range of the moderate class. Likewise, the mean CEC of the soil, which is an effective indicator of soil fertility and quality, was in the moderate class of 12–25 cmol kg^−1^^29^. The CEC was found to be highly correlated with clay (*r* = *0.76 P* < *0.01*) content and organic carbon (*r* = *0.54 P* < *0.05*) significantly. This result implies that CEC variations may accord with clay and carbon distribution. Most soil characteristics have a high coefficient of variations (CV) of > 35%, implying a wide variability of the soils in the study area.

**Table 2:** Summary statistics of selected attributes and heavy metals in the urban soils.

Soil variable	Max	Min	Mean	SD	CV
Clay (g kg^−1^)	610	100	348	15.8	45.5
Silt (g kg^−1^)	530	50	335	92.5	27.6
Sand (g kg^−1^)	850	110	307	196.3	58.6
pH	8.1	7.4	7.7	0.18	2.2
OC (%)	3.1	0.8	2.0	1.4	71.2
CCE (%)	35.0	5.5	23.4	11.8	50.4
ACC (%)	14.2	1.3	10.3	5.3	51.9
CEC (cmol kg^−1^)	35.7	10.1	22.8	6.3	27.6
Available P (mg kg^−1^)	18.6	8.8	12.8	5.8	45.4
Total N (%)	0.51	0.05	0.28	0.18	58.1
EC (dS m^−1^)	6.62	0.7	1.8	1.7	98.5
SAR	5.65	2.8	3.9	2.1	51.2
Cl^−1^(meql^−1^)	3.32	1.1	2.85	2.7	96.1
HCO_3_^–1^(meql^−1^)	1.7	0.57	1.65	1.3	81.2
Ca^2+^ (meql^−1^)	2.5	0.82	1.8	1.3	75.2
Mg^2+^ (meql^−1^)	1.7	0.35	0.92	0.58	63.4
K^+^(meql^−1^)	0.46	0.16	0.21	0.14	68.3
Na^+^ (meql^−1^)	3.8	0.82	2.4	2.3	95.8
Zn (mg kg^−1^)	55.35	184.87	80.22	16.86	21.1
Cu (mg kg^−1^)	22.91	121.5	38.74	6.98	18.2
Cd (mg kg^−1^)	0.48	1.54	0.88	0.47	53.4
Pb (mg kg^−1^)	29.67	122.13	57.98	30.62	52.8
Ni (mg kg^−1^)	30.65	99.55	53.44	12.75	23.3

*OC* organic carbon, *CCE* calcium carbonate equivalent, *ACC* active calcium carbonate, *CEC* cation exchange capacity, *EC* electrical conductivity, *SAR* sodium adsorption ratio.

### Soil heavy metals

The concentrations of Zn, Cu, Cd, Pb, and Ni varied in the ranges of 55.4–185, 22.9–121.5, 0.49–1.59, 29.1–122.1, and 21.8–99.5 mg kg^−1^, respectively (Table 2). Their mean concentrations descended in the order of Zn (80.2 mg kg^−1^) > Pb (58 mg kg^−1^) > Ni (55.4 mg kg^−1^) > Cu (38.8 mg kg^−1^) > Cd (0.88 mg kg^−1^). In most soil samples, these ranges are comparable with data reported for other urban soils around the world—e.g. Ref.^30^ in Poland, Ref.^31^ in China, and Ref.^32^ in Greece. The values of Cd, Cu, and Zn were below their acceptable ranges as per the international standards^4^ in all soil samples. Nonetheless, the Pb and Ni contents were higher than their acceptable ranges in 13.1% and 17.4% of the samples, respectively. Furthermore, the concentrations of the five elements were higher than their background values in all urban soil samples. This difference was considerable for Cd, Pb, and Ni. The heavy metals had CV in the order of Cd (53%) > Pb (51%) > Ni (46%) > Zn (21%) > Cu (18%). This CV variation implies great variations in Cd, Pb, and Ni, which is linked to anthropogenic activities^33^. The background values of the metals, estimated by the median absolute deviation method^10,14^, were 52.3, 18.7, 0.45, 29.1, and 30.8 mg kg^−1^ for Zn, Cu, Cd, Pb, and Ni, respectively.

**Figure 2:**
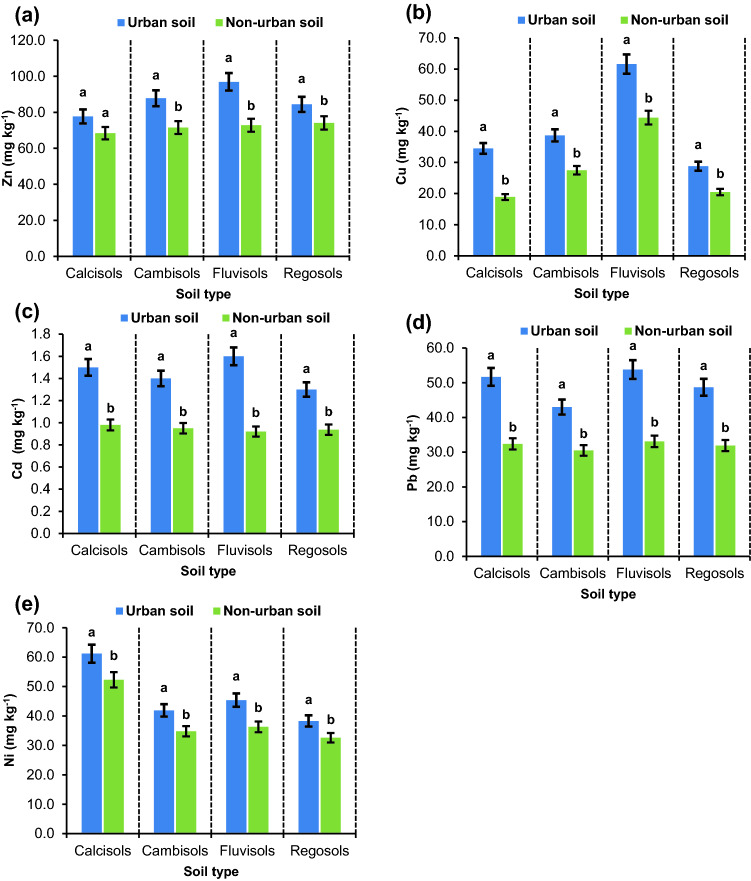
The comparison of the mean values of Zn (**a**), Cu (**b**), Cd (**c**), Pb (**d**), and Ni (**e**) between urban and non-urban soils in different soil types. Different letters indicate significant differences in metal content within each soil type at P < 0.05 confidence interval.

We compared the concentrations of the heavy metals between urban and non-urban soils and found significant increases in the concentration of the metals in most soil types (Fig. 2). The urban soils had 17–36%, 14–21%, 41–70%, 43–69%, and 13–24% higher Zn, Cu, Cd, Pb, and Ni contents than the non-urban soils. The effluent and waste entry from multiple food processing and storage units, dying plants, metal plating facilities, and plastic production in close proximity of the study area is believed to be the reason for the high concentration of these trace elements. Research in various parts of the world, e.g., Ref.^34^ in India, Ref.^35^ in Brazil, and Ref.^36^ in China, has documented that the facilities have introduced significant quantities of heavy metals to soils. However, traffic and agrochemicals also play a key role in the accumulation of heavy metals in this region^10^.

### Soil pollution indices

**Figure 3:**
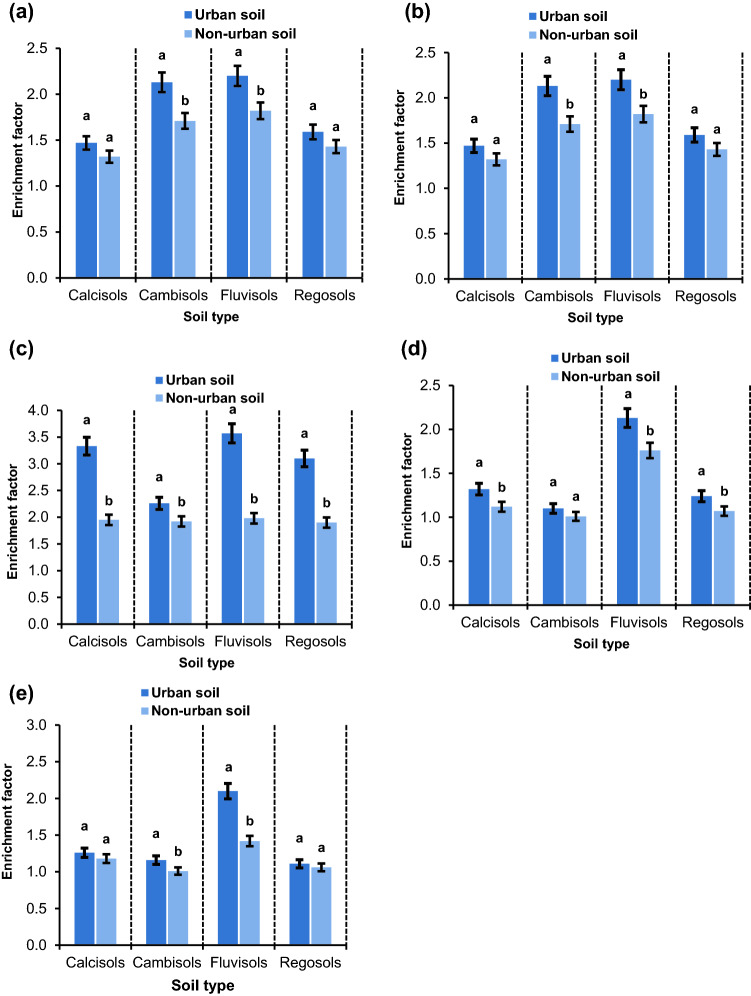
The comparison of the mean enrichment factor of Zn (**a**), Cu (**b**), Cd (**c**), Pb (**d**), and Ni (**e**) between urban and non-urban soils in different soil types. Different letters indicate significant differences in enrichment factor within each soil type at P < 0.05 confidence interval.

The pollution ranges of Zn, Cu, Cd, Pb, and Ni were assessed by calculating enrichment factor (EF), pollution index (PI), and ecological risk for each metal (E_i_) and for all metals (ER). The EF values were in ranges of 1.47–2.13, 1.1–2.2, 2.3–3.6, 1–2.13, and 1.1–2.1 for Zn, Cu, Cd, Pb, and Ni whereas they were in ranges of 1.3–1.8, 1–1.6, 1.9–2.2, 1–1.8, and 1–1.4 in the non-urban soils, respectively. The mean comparisons reveal an increase of 12–25% for Zn, 9–37% for Cu, 18–82% for Cd, 8–22% for Pb, and 7–48% for Ni in the urban soils versus the non-urban soils (Fig. 3). These increases upsurged the EF severity class from minimal enrichment (EF < 2) in the non-urban soils to moderate enrichment (2 ≤ EF < 5) in the urban soils. The shift in enrichment class occurred in 25% of the samples for Cu, 33% of the samples for Ni, 50% of the samples for Zn, 62% of the samples for Pb, and 100% of the samples for Cd. As a result, urban activities resulted in the enrichment of heavy metals in the order of Cd > Pb > Zn > Ni > Cu. These findings are comparable to the results reported by^37^ and^12^. The highest EF for all five elements was observed in the Fluvisols soil type, reflecting that this soil type had been exposed to element pollution induced by urban activities to a greater extent than the other soil types. In a study on the pollution potential of four soil types in Central Greece, Ref.^38^ reported different ranges of element pollution across different soil types.

As shown in Table 3, the mean PI of the urban soils decreased in the order of Cd (1.97) > Pb (1.89) > Ni (1.86) > Cu (1.73) > Zn (1.51). Mean PI for non-urban soils followed the order Cd (1.5) > Zn (1.4) > Cu (1.33) > Pb (1.31) > Ni (1.29). Nearly 7% and 16% of the urban soils showed moderate pollution (MP, PI = 2–3) and high pollution classes (HP, PI > 3) of PI for Cd and 39% and 4% showed the MP and HP class of PI for Pb, respectively. However, the PI class was low pollution (PI = 1–2) for all soil samples and soil types in the non-urban soils. The results on the pollution index indicate a widespread intensification of soil pollution in urban soils across all studied heavy metals.

**Table 3:** The level and terminology of PI and Ei of the analyzed heavy metals in urban and non-urban soils.

Parameter	Urban soil					Non-urban soil				
	Zn	Cu	Cd	Pb	Ni	Zn	Cu	Cd	Pb	Ni
**Pollution index (PI)**										
Min	1.04	1.02	1.08	1.1.05	1.02	1.03	1.01	1.03	1.02	1.01
Max	3.5	5.4	3.42	3.4	4.21	1.59	1.97	2.02	1.41	1.39
Mean	1.51	1.73	1.96	1.89	1.86	1.4	1.33	1.5	1.31	1.29
SD	0.4	0.81	0.71	0.62	1.1	0.22	0.45	0.33	0.31	0.42
**%Class of total samples**										
LP	89.8	81.1	76.8	56.5	65.2	100	100	92	100	100
MP	7.3	14.5	7.3	39.1	11.6			8		
HP	2.9	4.4	15.9	4.4	23.2					
**Single risk factor (Ei)**										
Min	1.04	5.11	32.5	5.23	5.12	1.03	4.93	31.2	4.1	4.3
Max	3.5	27.12	102.67	16.99	21.06	1.59	9.87	61.3	5.6	9.3
Mean	1.51	8.65	58.73	9.46	10	1.4	6.65	39.2	5.4	5.8
SD	0.4	4.1	21.2	3.1	4.9	0.22	3.2	12.7	2.4	2.5
**%Class of total samples**										
LR	100	100	36.7	100	100		100	90	100	100
MR			46.8					10		
CR			16.5							

PI; *LP* low pollution (1 < PI ≤ 2), *MP* moderate pollution (2 < PI ≤ 3), *HP* high pollution (PI > 3).

Ei; *LR* low risk (Ei ≤ 40), *MR* moderate risk (40 < Ei ≤ 80), *CR* considerable risk (80 < Ei ≤ 160).

Ecological risk, E_i_ was similarly found to be significantly higher in the urban soils than in the non-urban soils, even though the concentration of all elements except Cd fell within the low-risk class (E_i_ ≤ 40) in both urban and non-urban soils (Table 3). The mean E_i_ for Cd was 58.7 (moderate-risk class) and 39.2 (low-risk class) in the urban and non-urban soils, respectively. This means that urban activities have enhanced the ecological risk class of Cd by one grade. Overall, Cd had the highest EF, PI, and E_i_ among all heavy metals and in all soil samples, indicating a greater risk potential by Cd than Zn, Cu, Pb, and Ni across the water-soil–plant-human domain. Elevated Cd pollution by anthropogenic activities has been widely reported in the literature^10,12,39^. Cadmium as a Group 1 carcinogen element^40^ can accumulate in plant tissue without exhibiting visual symptoms. Therefore, Cd generally transfers from soil to the food chain covertly. Cadmium pollution can also influence soil quality and reduce crop yields and grain quality^3^.

**Figure 4:**
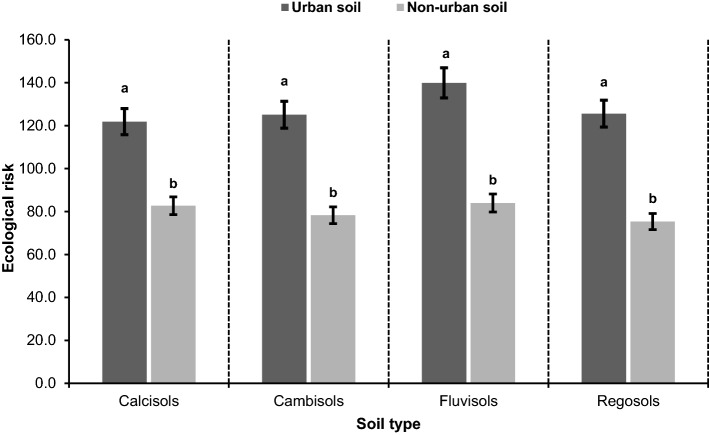
The comparison of the mean ecological risk of selected heavy metals between urban and non-urban soils in different soil types. Different letters indicate significant differences in ecological risk within each soil type at P < 0.05 confidence interval.

Similar to EF, PI, and E_i_, the mean ER was significantly elevated in all urban soil types than the non-urban soils (Fig. 4). Among different soil types, the ER magnitude was in the order of Fluvisols (66.6%) > Regosols (66.1%) > Cambisols (59.8%) > Calcisols (47%). These results indicate that Fluvisols carry a higher ecological risk potential for heavy metal accumulations than other soil types. In the study region, Fluvisols due to higher fertility and productivity are subject to more intense and extensive agronomic operations than other soil types^13^. Heavy application of agrochemicals (e.g., pesticides, herbicides, insecticides, and chemical fertilizers), accelerate the heavy metal input to the Fluvisols. Widespread application of nitrogen fertilizers and subsequent reduction in average soil pH markedly increases the solubility of certain heavy metals (e.g., Zn, Cu, Cd) which can be another factor increasing the ecological risk of heavy metal contamination in Fluvisols^41^. In addition, these Fluvisols are located on the margin of open urban wastewater channels, which are sometimes used for irrigation. A combination of mentioned processes can be implicated for higher ER of Fluvisols than that of other soil types as for BF, PI, and E_i_.

### The concentration of heavy metals in corn roots and grains

Table 4 presents the minimum, maximum, mean, and standard deviation of the metals in corn roots and grains. The concentrations of Zn, Cu, Cd, Pb, and Ni in the corn roots were in ranges of 4.3–21, 3.2–12.6, 0.4–0.74, 0.2–0.84, and 0.29–2.8 mg kg^−1^ with means of 9.9, 6.4, 0.55, 0.4, and 1.1 mg kg^−1^, respectively. The minimum, maximum, and mean values of these metals in the corn grains were 3.3, 16.6, and 7.9 mg kg^−1^ Zn, 2.4, 10.1, and 5.1 mg kg^−1^ Cu, 0.08, 0.15, and 0.11 mg kg^−1^ Cd, 0.05, 0.26, and 0.13 mg kg^−1^ Pb, and 0.06, 0.2, and 0.12 mg kg^−1^ Ni, respectively. Metal concentration was in the order of Zn > Cu > Ni > Cd > Pb in the roots, partially differing from that of the grain—Zn > Cu > Pb > Ni > Cd. Heavy metals concentrations observed in the corn roots and grains are almost comparable with those reported by^42^ in China and^43^ in Peru.

**Table 4:** Summary statistical attributes of the concentration of heavy metals in corn root (R) and grain (G) along with their BCF and TF.

Parameter	Min	Max	Mean	SD
Root concentration (mg kg^−1^)				
R-Zn	4.25	21.25	9.95	4.38
R-Cu	3.17	12.58	6.44	2.28
R-Cd	0.4	0.74	0.55	0.08
R-Pb	0.2	0.84	0.42	0.19
R-Ni	0.29	2.82	1.1	0.81
Grain concentration (mg kg^−1^)				
G-Zn	3.3	16.6	7.87	3.4
G-Cu	2.4	10.1	5.08	1.8
G-Cd	0.08	0.15	0.11	0.02
G-Pb	0.05	0.26	0.13	0.05
G-Ni	0.06	0.2	0.12	0.04
Bioconcentration factor (BCF)				
BCF-Zn	0.071	0.177	0.121	0.027
BCF-Cu	0.104	0.234	0.173	0.025
BCF-Cd	0.41	0.92	0.68	0.13
BCF-Pb	0.005	0.011	0.007	0.001
BCF-Ni	0.009	0.025	0.016	0.004
Translocation factor (TF)				
TF-Zn	0.749	0.943	0.795	0.023
TF-Cu	0.722	0.851	0.789	0.03
TF-Cd	0.171	0.222	0.199	0.01
TF-Pb	0.089	0.629	0.313	0.077
TF-Ni	0.005	0.216	0.15	0.056

The accumulation of heavy metals in the edible parts of corn is of higher importance. In the present study, the concentrations of these metals were lower than the acceptable level in the corn grains based on international references^44^. So, the consumption of corns grown in the regions should not threaten human and animal health in the short term, but caution should be exercised in their long-term consumption because some of these elements, especially Cd and Pb, which have long decomposition half-lives, gradually accumulate in body organs, especially in kidneys and livers^45^. Besides, the ratio of Zn, Cu, Cd, Pb, and Ni of the corn grain to their acceptable standard concentration, known as the pollution index of crop heavy metals, Ref.^12^ was lower than 0.7 for most corn samples, indicating the unpolluted risk class.

The mean concentrations of Cd, Pb, and Ni were 5, 3.1, and 9.2 times as great in the corn roots as in their grains. This observation exhibits a notable phytoremediatory function of corn roots through restriction of radial translocation of heavy metals to the xylems and eventually into the grains. A similar trend of heavy metal accumulation in different plant organs has been reported in previous observations^46,47^. Based on Kabata-Pendias^4^ and Adriano^22^, plant cells can use the defensive tools of the roots to cope with heavy metals, especially Cd and Pb—highly toxic metals to plant cytosols. Accordingly, plant cells can fix these elements in the root system by such approaches as precipitating on cell walls, storing in vacuoles, and/or chelating by phytochelatins, thereby alleviating their toxic effects and inhibiting their translocation to plant shoots. For Zn, Cu, and Cd metals, a significant correlation was observed between their concentration in corn roots and grains. But, a less significant correlation (P < 0.05) was recorded for Pb and Ni with r = 0.52 and r = 0.38 (Table 5). These correlations indicate that a major part of Zn, Cu, and Cd in the corn grains may have been mobilized by their soil-root system, while a combination of atmospheric precipitation and root-soil system may be implicated for Pb and Ni^4^.

**Table 5:** Pearson correlation coefficients between heavy metals concentration of (in soil and corn root and grain) with selected soil properties.

Variables	pH	EC	OC	CEC	CCE	Cd	Ni	Pb	Zn	Cu	R-Cd	R-Ni	R-Pb	R-Zn	R-Cu	G-Cd	G-Ni	G-Pb	G-Zn	G-Cu
pH	**1**	− 0.382*	0.363*	0.056	0.108	− 0.037	0.013	0.155	0.088	0.105	0.099	− 0.190	0.094	0.111	0.137	0.061	− 0.063	0.124	0.114	0.126
EC	− 0.382*	**1**	0.099	0.043	0.235*	− 0.208	− 0.050	− 0.158	− 0.122	− 0.042	− 0.234*	− 0.262*	− 0.183	− 0.050	0.078	− 0.211	0.216	− 0.236*	− 0.053	0.066
OC	0.368*	0.099	**1**	0.335*	0.281*	0.003	− 0.123	− 0.088	0.175	0.311*	0.099	− 0.002	− 0.121	0.266*	0.317*	0.091	0.085	− 0.075	0.266*	0.300*
CEC	0.056	0.043	0.335*	**1**	0.20*	0.311*	− 0.120	0.038	0.058	− 0.007	0.289*	− 0.118	0.003	0.029	0.051	**0.322**	− 0.131	− 0.022	0.017	0.047
CCE	0.108	0.235*	0.281*	0.20*	**1**	0.36*	− 0.070	− 0.26*	− 0.070	− 0.106	0.38*	− 0.135	**− 0.27**	− 0.051	0.041	**0.36**	− 0.087	− 0.25*	− 0.057	0.016
Cd	− 0.037	− 0.208	0.003	0.311*	− 0.36*	**1**	− 0.010	0.195	0.092	0.338*	0.901**	− 0.156	0.212	− 0.104	0.175	0.816**	− 0.177	0.171	− 0.121	0.175
Ni	0.013	− 0.050	− 0.123	− 0.120	− 0.070	− 0.010	**1**	− 0.118	0.181	0.054	− 0.036	0.463*	− 0.107	0.266*	0.070	− 0.075	0.34*	− 0.119	0.263*	0.076
Pb	0.155	− 0.158	− 0.088	0.038	− 0.26*	0.195	− 0.118	**1**	− 0.182	− 0.124	0.183	0.047	0.68***	− 0.224	− 0.173	0.221	− 0.013	0.54***	− 0.232	− 0.187
Zn	0.088	− 0.122	0.175	0.058	− 0.070	0.092	0.181	− 0.182	**1**	0.327*	0.119	− 0.120	− 0.138	0.906**	0.247*	0.051	− 0.026	− 0.128	0.901**	0.229
Cu	0.105	− 0.042	0.311*	− 0.007	− 0.106	0.338*	0.054	− 0.124	0.327*	**1**	0.305*	− 0.144	− 0.155	0.120	0.835**	0.158	− 0.083	− 0.207	0.121	0.820**
R-Cd	0.099	− 0.234*	0.099	0.289*	0.38*	0.901**	− 0.036	0.183	0.119	0.305*	**1**	− 0.093	0.193	− 0.029	− 0.189	0.945**	− 0.083	0.159	− 0.045	0.186
R-Ni	− 0.190	0.262*	− 0.002	− 0.118	− 0.135	− 0.156	0.463*	0.047	− 0.120	− 0.144	− 0.093	**1**	0.031	− 0.054	− 0.083	− 0.040	0.38*	0.013	− 0.061	− 0.096
R-Pb	0.094	− 0.183	− 0.121	0.003	− 0.27*	0.212	− 0.107	0.68***	− 0.138	− 0.155	0.193	0.031	**1**	− 0.205	− 0.217	0.230	0.005	0.52*	− 0.210	− 0.233
R-Zn	0.111	− 0.050	0.266*	0.029	− 0.051	− 0.104	0.266*	− 0.224	0.906**	0.120	− 0.029	− 0.054	− 0.205	**1**	0.137	− 0.048	0.015	− 0.171	0.998**	0.117
R− Cu	0.137	0.078	0.317*	0.051	0.041	0.175	0.070	− 0.173	0.247*	0.835**	0.189	− 0.083	− 0.217	0.137	**1**	0.087	0.078	− 0.267*	0.139	0.994**
G-Cd	0.061	− 0.211	0.091	0.322*	0.36*	0.816**	− 0.075	0.221	0.051	0.158	0.945**	− 0.040	0.230	− 0.048	0.087	**1**	− 0.060	0.217	− 0.066	0.078
G-Ni	− 0.063	0.216	0.085	− 0.131	− 0.087	− 0.177	0.34*	0.463*	− 0.026	− 0.083	− 0.083	0.38*	0.005	0.015	0.078	− 0.060	**1**	− 0.026	0.010	0.061
G-Pb	0.124	− 0.236*	− 0.075	− 0.022	− 0.25*	0.171	− 0.119	0.54***	− 0.128	− 0.207	0.159	0.013	**0.905**	− 0.171	− 0.267*	0.217	− 0.026	**1**	− 0.177	− 0.271*
G-Zn	0.114	− 0.053	0.266*	0.017	− 0.057	− 0.121	0.263*	− 0.232	0.901**	0.121	− 0.045	− 0.061	− 0.210	0.998**	0.139	− 0.066	0.010	− 0.177	**1**	0.118
G-Cu	0.126	0.066	0.300*	0.047	0.016	0.175	0.076	− 0.187	0.229	0.820**	− 0.186	− 0.096	− 0.233	0.117	0.994**	0.078	0.061	− 0.271*	0.118	**1**

*, **, and *** indicate the significance of correlation at 0.05, 0.01, and 0.001 confidence intervals, respectively.

### Bioconcentration factor (BCF) and translocation factor (TF)

The values of BCF and TF were calculated considering the concentrations of heavy metals in the soil, corn roots, and corn grains (Table 4). The mean BCF of the elements was in the descending order of Cd (0.68) > Cu (0.17) > Zn (0.12) > Ni (0.02) > Pb (0.01). This implies that Cd, and to a smaller extent Cu is taken up by corn roots from the soil more readily, but Pb and Ni are less absorbable. These results are consistent with the reports of^48^ and^46^. The greater value of BCF-Cd may be related to a combination of the specific factors e,g., Cd concentration and chemistry, as well as soil characteristics (e.g., soil texture, pH, and calcium carbonate content)^4^. As was already discussed, the examined soils were characterized by high alkaline (pH = 7.4–8.1) and calcareous properties (CCE = 5.5–35%) with a high concentration of Soluble salts (EC = 0.7–6.6 dS m^−1^). These characteristics can result in the formation of complex Cd ions, especially CdOH^+^, CdCl_2_^0^, CdCl^+^, CdSO_4_^0^, and CdHCO_3_^+^^4,22^. These ions are plant-available, resulting in a further increase in Cd BCF. Regarding Ni and Pb, the alkaline and calcareous properties of the soils may have motivated insoluble compounds such as NiHCO_3_^+^ and NiCO_3_^0^ (for Ni) and Pb(OH)_2_, PbCO_3_, PbSO_4_, and PbO (for Pb)^4,22^. These compounds cannot be uptake by plant roots, which may have resulted in a significant decrease in the BCF of these metals versus the other analyzed elements.

Like BCF, the heavy metals had TF of < 1, which is similar to some previous studies that reported TF < 1 for the translocation of heavy metals from roots to grains for crops such as wheat, corn, and rice^49–51^. The mean TF of the metals decreased in the order of Zn (0.8) > Cu (0.78) > Pb (0.21) > Cd (0.2) > Ni (0.15). This implies that Zn and Cu are translocated from roots to grains readily, about four times as great as the other metals, while Ni, Cd, and Pb are translocated in smaller concentrations.

**Figure 5:**
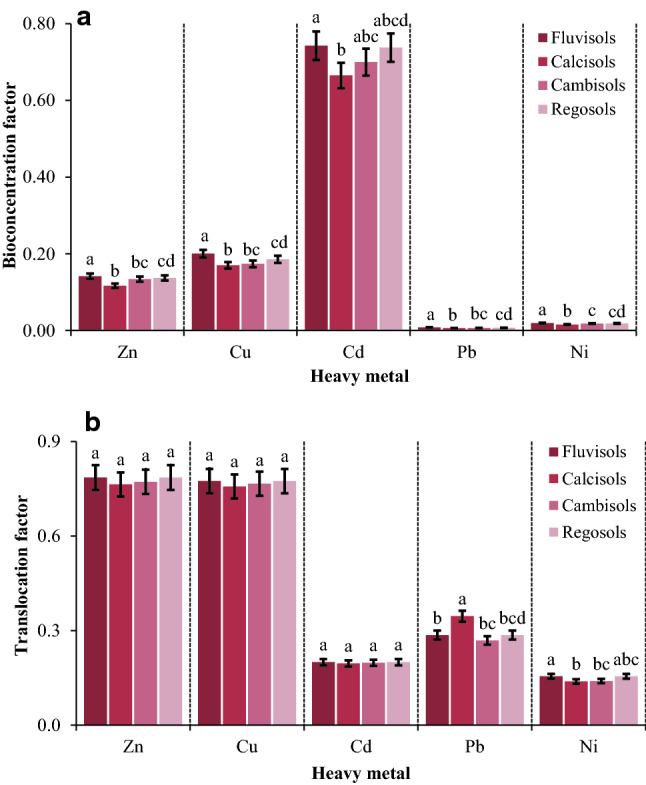
Effect of soil type on the mean bioconcentration factor (**a**) and translocation factor (**b**) of selected heavy metals in urban soils. Different letters indicate significant differences in bioconcentration and translocation factors among soil types for each metal at P < 0.05 confidence interval.

The comparison of BCF and TF of Cd showed that less than 30% of Cd, on average, accumulated in the corn roots were translocated to the grains. This states that Cd is immobilized by various mechanisms before it can find its way into the grains. Some of the important mechanisms include (i) the antagonistic effects of Cd with other equivalent elements, especially Zn, Fe, and Ca, in the vascular system of corn, which reduces its mobility in the corn root-stem-grain system^22^, (ii) Cd sequestration in active exchange sites on the cell wall in the corn root-stem pathway^10^, and (iii) the binding of Cd with some specific compounds, e.g., phytochelatins of root vacuoles, which immobilizes it before its translocation to grains^4,22^. Lin and Aarts^52^ remarked that Cd mostly tends to be trapped in root vacuoles, which reduces its translocation to the upper parts of the plants. In general, it was found that corn plants have a high potential to absorb and accumulate Cd in their roots and Zn in their grains, which is consistent with previous studies^41^. For the majority of heavy metals, the values of BCF and TF in different soil types were in the order of Fluvisols > Regosols > Cambisols > Calcisols, indicating that the great variety of soil types for the uptake and translocation of heavy metals in the soil-root-grain of the corn (Fig. 5).

### Multivariate analysis of metals concentration and accumulation in root and grain corn

The relationship of the heavy metals concentration in root and grain of cron with soil characteristics was explored by multivariate regression models. These models can be used as an easy tool to predict the concentration of heavy metals and the risk of their accumulation in various crops^10^. Significant relationships were observed between Zn, Cu, Cd, and Pb concentrations in soil and associated concentrations in corn root and shoots(Table 6) while the relationship was non-significant for Ni. This implies that a considerable value of corn Ni might be attributed to non-soil sources—e.g., atmospheric depositions, or metal-bound dust. Among different properties of soil, CCE, CEC, and concentration of Cd and Cu were the key factors influencing Cd accumulation in the roots and grain of corn, accounted for 81% and 67%, of the variance, respectively. For Pb, the values of soil CCE, Pb, and Cd had a significant impact on Pb-root and Pb-grain, accounted for 94% and 63%, of the variance, respectively. The models indicated that the predicted Cd content in the root and grain of corn increased with an increase in soil Cd content, calcium carbonate, CEC. However, an increase in soil calcium carbonate and Cd concentration decreased the predicted Pb content in the corn root and grain. As previously discussed, the positive and negative impact of calcium carbonate on corn Cd and Pb, respectively, is likely due to the formation of the complexes of Cd with carbonate which is readily uptake by plant roots, and the formation of insoluble compounds of Pb which cannot be absorbed by plant roots^4^. Besides, the regression model Pb showed an antagonistic interaction between soil Cd and Pb which might have resulted in a decrease in Pb uptake by roots crop^22^.

**Table 6:** Multiple regression models for predicting the concentration of heavy meals in the root and grain of corn.

Dependent variable	Independent variable	Regression models	R^2^	Sig
Zn-Root	Soil Zn, OM, Soil Ni	Zn _(Root)_ = 5.49 + 0.48 OM + 0.00015 Ni _(soil)_—0.18 Zn _(soil)_	0.848	0.001
Cu-Root	Soil Cu, OM, Soil Zn	Cu _(Root)_ = 2.28 + 0.31 OM – 0.0054 Zn _(soil)_ + 0.1 Cu _(soil)_	0.723	0.001
Cd-Root	Soil Cd, CEC, EC, CCE, Soil Cu	Cd _(Root)_ = 0.35 + 0.00029 CEC – 0.00017 EC – 0.000035 CCE + 0.23 Cd _(soil)_ + 0.000013 Cu _(soil)_	0.814	0.001
Pb-Root	Soil Pb and CCE	Pb _(Root)_ = -0.048 – 0.0025 CCE + 0.0095 Pb _(soil)_	0.935	0.001
Ni-Root	Soil Ni and EC	Log Ni _(Root)_ = -0.75 + 0.19 log EC + 0.37 log Ni _(soil)_	0.301	ns
Zn-Grain	Soil Zn, OM, Soil Ni	Zn _(Grain)_ = -4.11 + 0.38 OM + 0.00015 Ni _(soil)_ + 0.14 Zn _(soil)_	0.839	0.001
Cu-Grain	Soil Cu and OM	Cu _(Grain)_ = 1.54 + 0.2 OM + 0.081 Cu _(soil)_	0.74	0.001
Cd-Grain	Soil Cd, CEC, CCE	Cd _(Grain)_ = 0.071 + 0.00022 CEC + 0.000014CCE + 0.039 Cd _(soil)_	0.67	0.001
Pb-Grain	Soil Pb, CCE, and Soil Cd	Pb _(Grain)_ = 0.016—0.00072 CCE – 0.0031 Cd _(soil)_ + 0.0023 Pb _(soil)_	0.63	0.01
Ni-Grain	Soil Ni and EC	Log Ni _(grain)_ = 0.0056 + 0.027 log EC + 0.037 log Ni _(soil)_	0.24	ns

Commonly, organic carbon was an important factor influencing Zn and Cu accumulation in corn root (capturing 85 and 72% of the variance, respectively) and grain (capturing 84 and 74% of the variance, respectively). This may be explained by the synthesis of organometallic complexes of Zn and Cu that enhance the availability of two metals and protect them from precipitation reactions, resulted in an increase in their uptake by crops^22,52^.

### Health risk assessment

#### Non-carcinogenic risk

Since corn grains have traditionally been a staple food for Iranians, the daily intake (DI), hazard quotient (HQ), and hazard index (HI) of heavy metals translocated by corn ingestion were calculated to different demographic groups, including children, adult women, and adult men (Table 7). The mean DI was varied in ranges from 0.0012 (Cd) to 0.084 (Zn), from 6.27 E−05 (Ni) to 0.004 (Zn), and from 4.78E−05 (Cd) to 0.0034 (Zn) for children, women, and men, respectively. So, Zn had the highest DI for all demographic groups. Nonetheless, the DI values are within the acceptable range for all metals and demographic groups according to WHO/FAO (2017). The comparison of the mean DI among the three demographic groups showed that DI of Zn, Cu, Cd, Pb, and Ni in children was 21, 20.7, 21.4, 20.4, and 20.7 and 24.7, 24.5, 25.1, 23.9, and 24.3 times of woman and men, respectively, which accords with previous research by^51^. These differences may be due to the different nutritional behaviors of the demographic groups. The result for higher DI of the children is related to the fact that although children consume lower volumes of food, their body weight is lower than that of the other groups^10^. These data also mean that the effects of an environmental threat, like heavy metals, by food consumption is not similar across different demographic groups.

**Table 7:** Summary statistical attributes of DI, HQ, and HI indifferent age groups.

Heavy metal	Children				Adult-female				Adult-male			
	Min	Max	Mean	SD	Min	Max	Mean	SD	Min	Max	Mean	SD
**Daily intake of heavy metals (DI)**												
Zn	0.035294	0.17754	0.084171	0.0365	0.001684	0.008469	0.004015	0.001741	0.001436	0.007221	0.003423	0.001485
Cu	0.025668	0.108021	0.054315	0.019174	0.001224	0.005153	0.002591	0.000915	0.001044	0.004394	0.002209	0.00078
Cd	0.000856	0.001604	0.001176	0.000168	4.08125E−05	7.65234E−05	5.61125E−05	8.01865E−06	0.0000348	0.00006525	4.7846E−05	6.83735E−06
Pb	0.000567	0.002781	0.001333	0.000558	2.7038E−05	0.00013264	6.3576E−05	2.6628E−05	0.000023055	0.0001131	5.42101E−05	2.27052E−05
Ni	0.00062	0.002096	0.001314	0.00039	2.9589E−05	9.99906E−05	6.26751E−05	1.86253E−05	0.00002523	0.00008526	5.34418E−05	1.58814E−05
**Hazard quotient (HQ)**												
Zn	0.117647	0.5918	0.280571	0.121666	0.005612	0.028229	0.013383	0.005803	0.004785	0.02407	0.011412	0.004948
Cu	0.064171	0.270053	0.135787	0.047936	0.030609	0.128814	0.06477	0.022865	0.0261	0.109838	0.055228	0.019497
Cd	0.855615	1.604278	1.176373	0.168107	0.040812	0.076523	0.056113	0.008019	0.0348	0.06525	0.047846	0.006837
Pb	0.014171	0.069519	0.033321	0.013956	0.00676	0.03316	0.015894	0.006657	0.005764	0.028275	0.013553	0.005676
Ni	0.00031	0.001048	0.000657	0.000195	0.001479	0.005	0.003134	0.000931	0.001262	0.004263	0.002672	0.000794
**Hazard index (HI)**												
	1.156738	2.3121	1.625068	0.213396	0.117455	0.232023	0.15319	0.025459	0.100152	0.197842	0.130492	0.021831

HQ for different metals was in the order of Cd > Zn > Cu > Pb > Ni for children, differing from that for adults (Cu > Cd > Pb > Zn > Ni). The values of HQ was < 1 for all metals, except for Cd, in all three demographic groups, reflecting the negligible risk of Zn, Cu, Pb, and Ni to cause non-cancerogenic diseases among corn consumers in the study region^53^. Similar to DI, HQ of the metals was the highest for children followed by women and men. So, children are more exposed to non-carcinogenic risks than adult females and males. Regarding Cd, HQ was > 1 in over 87% of the samples, implying the low non-carcinogenic risk of this metal for corn-consuming children in the study region^53^. Rapidly developing children’s nervous system are highly sensitive to environmental factors, including heavy metals, so even a relatively low concentration of Cd in children’s blood may irreversibly affect their mental growth and functioning^54^.

The highest HI was observed in children (min = 1.16, max = 2.31, mean = 1.63) followed by women and men which was similar to the found pattern of HQ (Table 7). These data show a moderate non-carcinogenic health risk (1 ≤ HI < 4) for corn-consuming local children. In contrast, the local adults, including both females and males, exhibited a low non-carcinogenic health risk (0.1 ≤ HI < 1) caused by corn grain consumption (USEPA, 2004). Similarly, a study in western Iran^55^ reported the HI values higher and lower than the acceptable levels for Cd, Pb, Zn, Cu, Ni, and Hg in grains and other cereals (corn, wheat, rice, peas, and beans) for children and adults, respectively. In all demographic groups, Cd and Cu had the highest contribution in HI followed by Zn and Pb. The contributions of these three elements in HI were 37–72.5%, 8.3–42%, and 8.7–27.2%, respectively. As a result, the highest HQ for the demographic groups consuming corn grains was related to Cd, so it had the highest impact on the HI value. Such a pattern of Cd may cause its long-term accumulation in vital organs, especially in kidneys and liver of corn-consuming individuals, thereby leading to severe damages in neural systems, skeletons, lungs, and enzymatic and immune systems^56,57^.

#### Carcinogenic risk

**Figure 6:**
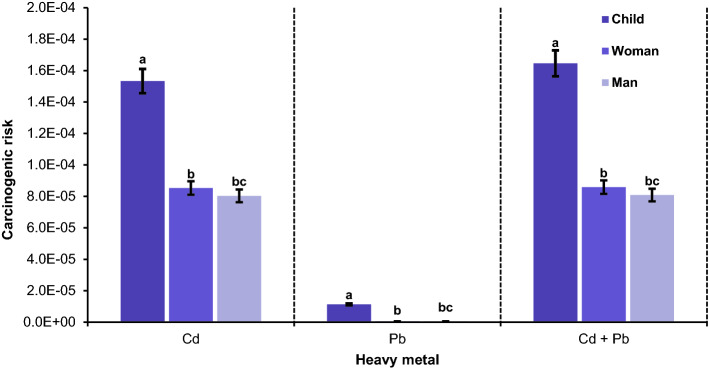
The comparison of the mean carcinogenic risk of Cd, Pb, and Cd + Pb among different population groups in urban soils. Different letters indicate significant differences among population groups in regard with each metal at P < 0.05 confidence interval.

Since the cancer slope factor was available for Cd and Pb, the carcinogenic risk (CR) of these two metals by corn grain consumption was calculated for different demographic groups. The values of CR of the two metals for children, women, and men were in ranges from 1.1E−04 to 1.5E−04, 6.21E−05 to 8.53E−05, and 5.84E−05 to 8.03E−05 for Cd and from 4.82E−06 to 1.13E−05, 2.33E−07 to 5.48E−07, and 2.19E−07 to 5.16E−07 for Pb, respectively. These data mean that CR is almost within the acceptable range (from 1.00E−04 to 1.00E−06) for corn-consuming adults in the region, but children are exposed to the low to moderate risk of cancer^53^. Regarding Cd, the mean CR was 1.40E−04, 8.53E−05, and 8.03E-5 for children, adult females, and adult males, respectively (Fig. 6). This implies that occurrences of 14 and 8 cases out of 1,000,000 of children and adults are exposed to the risk of cancer caused by Cd. The lead had lower CR than Cd and its risk of cancer was one child and five adults cases out of 1,000,000 individuals. The comparison of these data shows that the risk of Cd-caused cancer through corn ingestion among different demographic groups was 13.5 for children and 155 times for adults higher than that caused by Pb in the region. This corroborates the findings of^41^ and^43^, who reported higher CR for Cd than for other metals taken up by grain consumption.

**Figure 7:**
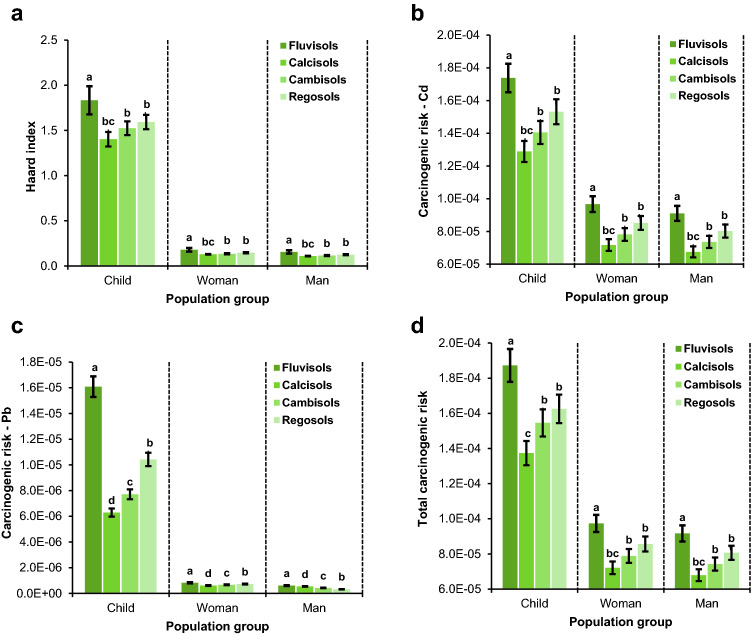
The effect of soil type on the mean hazard index (**a**) and carcinogenic risk of Cd (**b**), Pb (**c**), and Cd + Pb in urban soils. Different letters indicate significant differences in carcinogenic risk within population groups at P < 0.05 confidence interval.

Total carcinogenic risk (TCR) for Cd and Pb was the highest for children (min = 1.20E−04, max = 2.30E−04, mean = 1.60E−04) followed by females (min = 6.24E−05, max = 1.20E−04, mean = 8.59E−05) and males (min = 5.88E−05, max = 1.10E−05, mean = 8.09E−05). Therefore, TCR for women (more than 86% of samples) and men (more than 95% of samples) are almost within the acceptable range (1.00E−04 to 1.00E−06), but considering the TCR data, children are exposed to a moderate level of carcinogenic risk (more one case of cancer after every 1,00,000 inhabitants). These findings are supported by the findings of^34^ and^58^ for the CR of Cd and Pb metals. The highest values of HI, CR-Cd, CR-Pb, and TCR were observed in Fluvisols (Fig. 7), showing that this soil type is more polluted than the other types and has a higher potential to cause cancerous and non-cancerous risks.

Our study provides insight into the urbanization impact on heavy metal contamination in the soil system to the food chain and the associated health risks across a range of soil types, metals, age, and gender groups. Our finding is expected to aid policymakers and NGOs with hands-on information regarding the state of heavy metal contamination and associated health risks in northwestern Iran. It should be acknowledged that further studies are required to fully understand the mechanisms of absorption, mobility, translocation, and accumulation of heavy metals in different parts of grains (roots, stems, leaves, and grains) using bioclinical models that are dependent on animal and human health. At present, the studies on the analyses of human health risk caused by heavy metals merly rely on thresholds and prediction models defined by the Environmental Protection Agency of the US. While the local data supporting the validity of those standards around the world is lacking.

## Conclusion

We determined pollution index (PI), enrichment factor (EF), ecological risk (ER), bioconcentration factor (BCF), and translocation factor (TF), as well as carcinogenic and non-carcinogenic risk models to assess the effect of urban activities on the pollution status of Zn, Cu, Cd, Pb, and Ni in soil system and its relationship with the health risk of humans in a calcareous-semi-arid region under corn cultivation with four different soil types. Compared to the control soil (non-urban soil), the mean EF for the analyzed soils significantly increased in the order of Cd > Pb > Zn > Ni > Cu. The value of ER was considerable in all urban soil samples, which was mainly related to Cd, whereas it was moderate in the control soil. So, urban activities have increased ER by one grade.The comparison of BCF and TF of the five metals in the soil-corn root-stem-grain pathways showed that the roots of this plant may act as a filter limiting the translocation of some metals, especially Cd, from the roots to the grains. For the corn-consuming children age group, HQ of Cd was higher than 1 in 87% of the samples and HI of the metals was higher than 1 in 100% of the samples, whereas they were associated with smaller values than those for adults. The CR caused by Cd and Pb and their combination (TCR) was over one case out of 100,000 for children, reflecting a moderate range of carcinogenic risk among local children, although they were in the acceptable range (from 1.00E−04 to 1.00E−06) for adults in most soil samples.

Overall, our study addressedthe level and extent of heavy metal contamination and associated health risks in the cultivated urban soils. Further, we quantified the relationship between soil type and heavy metals patterns to advance the ecological protection and restoration of the study region.The future research will be centered around additional data collection and simulation of the absorption and translocation of heavy metals in the soil-grain roots-stems-grains system based on soil type. The development of bioclinical models for these metals in the food chain can contribute to alleviating the risk of heavy metals and controlling the food safety for the enhanced livelihood of local citizens.

## References

[1] Edmondson JL, Davies ZG, McHugh N, Gaston KJ, Leake JR (2012). Organic carbon hidden in urban ecosystems. Sci. Rep..

[2] Gu Y-G, Gao Y-P (2018). Bioaccessibilities and health implications of heavy metals in exposed-lawn soils from 28 urban parks in the megacity Guangzhou inferred from an in vitro physiologically-based extraction test. Ecotoxicol. Environ. Saf..

[3] Mamehpour, N., Rezapour, S. & Ghaemian, N. Quantitative assessment of soil quality indices for urban croplands in a calcareous. *Geoderma* **382**, 114781 (2021).

[4] Kabata-Pendias, A. *Trace elements in soils and plants*. (CRC press, 2000).

[5] Nations, U. Revision of world urbanization prospects. *United Nations: New York, NY, USA* (2018).

[6] Rate AW (2018). Multielement geochemistry identifies the spatial pattern of soil and sediment contamination in an urban parkland, Western Australia. Sci. Total Environ..

[7] Tang J (2019). Diagnosis of soil contamination using microbiological indices: A review on heavy metal pollution. J. Environ. Manage..

[8] USEPA. (United States Environmental Protection Agency Philadelphia (PA), 2007).

[9] Chen H, Teng Y, Lu S, Wang Y, Wang J (2015). Contamination features and health risk of soil heavy metals in China. Sci. Total Environ..

[10] Rezapour, S., Atashpaz, B., Moghaddam, S. S. & Damalas, C. A. Heavy metal bioavailability and accumulation in winter wheat (Triticum aestivum L.) irrigated with treated wastewater in calcareous soils. *Sci. Total Environ.* **656**, 261–269 (2019). 10.1016/j.scitotenv.2018.11.288 30504026

[11] Gąsiorek M, Kowalska J, Mazurek R, Pająk M (2017). Comprehensive assessment of heavy metal pollution in topsoil of historical urban park on an example of the Planty Park in Krakow (Poland). Chemosphere.

[12] Huang Y (2018). Heavy metal pollution and health risk assessment of agricultural soils in a typical peri-urban area in southeast China. J. Environ. Manage..

[13] WRB, I. W. G. World reference base for soil resources 2014, update 2015. *World Soil Resources Reports 106* (2015).

[14] Rezapour S, Samadi A, Kalavrouziotis IK, Ghaemian N (2018). Impact of the uncontrolled leakage of leachate from a municipal solid waste landfill on soil in a cultivated-calcareous environment. Waste Manage..

[15] Yeilagi S, Rezapour S, Asadzadeh F (2021). Degradation of soil quality by the waste leachate in a Mediterranean semi-arid ecosystem. Sci. Rep..

[16] Sparks, D. L., Page, A. L., Helmke, P. A. & Loeppert, R. H. *Methods of soil analysis, part 3: Chemical methods*. Vol. 14 (John Wiley & Sons, 2020).

[17] Chapman, H. Cation‐exchange capacity. *Methods of Soil Analysis: Part 2 Chemical and Microbiological Properties* **9**, 891–901 (1965).

[18] Drouineau G (1942). Dosage rapide du calcaire actif du sol: Nouvelles données sur la separation et la nature des fractions calcaires. Ann. Agron.

[19] Bremner, J. & Mulvaney, C. Nitrogen-total in: Page, al ed. Methods of soil analysis. Part 2: Chemical and microbiological properties, 595–624. *Am. Soc. Agron., Madison, WI, USA* (1982).

[20] Olsen S, Sommers L (1982). & Page, A.

[21] Richards, L. A. *Diagnosis and improvement of saline and alkali soils*. Vol. 78 (LWW, 1954).

[22] Adriano, D. C. *Trace elements in terrestrial environments: biogeochemistry, bioavailability, and risks of metals*. Vol. 860 (Springer, 2001).

[23] Bühl, A. *SPSS 16: Einführung in die moderne Datenanalyse*. Vol. 7332 (Pearson Deutschland GmbH, 2008).

[24] Hakanson L (1980). An ecological risk index for aquatic pollution control A sedimentological approach. Water Res..

[25] Sutherland R (2000). Bed sediment-associated trace metals in an urban stream, Oahu Hawaii. Environ. Geol..

[26] EPA, A. Risk Assessment Guidance for Superfund. Volume I: Human Health Evaluation Manual (Part E, Supplemental Guidance for Dermal Risk Assessment). (EPA/540/R/99, 2004).

[27] DoE, U. The risk assessment information system (RAIS). *Argonne, IL: US Department of Energy’s Oak Ridge Operations Office (ORO)* (2011).

[28] Rezapour S, Samadi A, Khodaverdiloo H (2012). Impact of long-term wastewater irrigation on variability of soil attributes along a landscape in semi-arid region of Iran. Environ. Earth Sci..

[29] Hazelton, P. & Murphy, B. *Interpreting soil test results: What do all the numbers mean?* , (CSIRO publishing, 2016).

[30] Mazurek R (2017). Assessment of heavy metals contamination in surface layers of Roztocze National Park forest soils (SE Poland) by indices of pollution. Chemosphere.

[31] Pan L (2018). A review of heavy metal pollution levels and health risk assessment of urban soils in Chinese cities. Environ. Sci. Pollut. Res..

[32] Argyraki A (2018). Environmental availability of trace elements (Pb, Cd, Zn, Cu) in soil from urban, suburban, rural and mining areas of Attica Hellas. J. Geochem. Explor..

[33] Rezapour S, Moazzeni H (2016). Assessment of the selected trace metals in relation to long-term agricultural practices and landscape properties. Int. J. Environ. Sci. Technol..

[34] Adimalla, N., Chen, J. & Qian, H. Spatial characteristics of heavy metal contamination and potential human health risk assessment of urban soils: A case study from an urban region of South India. *Ecotoxicol. Environ. Saf.* **194**, 110406 (2020). 10.1016/j.ecoenv.2020.110406 32151868

[35] Penteado, J. O. *et al.* Health risk assessment in urban parks soils contaminated by metals, Rio Grande city (Brazil) case study. *Ecotoxicol. Environ. Saf.* **208**, 111737 (2021). 10.1016/j.ecoenv.2020.111737 33396065

[36] Pecina, V. *et al.* Human health and ecological risk assessment of trace elements in urban soils of 101 cities in China: A meta-analysis. *Chemosphere*, 129215 (2020). 10.1016/j.chemosphere.2020.129215 33359981

[37] Adamcová D (2017). Environmental assessment of the effects of a municipal landfill on the content and distribution of heavy metals in Tanacetum vulgare L. Chemosphere.

[38] Golia E, Dimirkou A, Mitsios I (2009). Heavy-metal concentration in tobacco leaves in relation to their available soil fractions. Commun. Soil Sci. Plant Anal..

[39] El-Hassanin AS, Samak MR, Abdel-Rahman GN, Abu-Sree YH, Saleh EM (2020). Risk assessment of human exposure to lead and cadmium in maize grains cultivated in soils irrigated either with low-quality water or freshwater. Toxicol. Rep..

[40] IARC, C. (Obtenido de monographs. iarc. fr: http://cort.as/-DxOC, 2018).

[41] Wang J (2019). Iron–manganese (oxyhydro) oxides, rather than oxidation of sulfides, determine mobilization of Cd during soil drainage in paddy soil systems. Environ. Sci. Technol..

[42] Mao C (2019). Human health risks of heavy metals in paddy rice based on transfer characteristics of heavy metals from soil to rice. CATENA.

[43] Román-Ochoa Y (2021). Heavy metal contamination and health risk assessment in grains and grain-based processed food in Arequipa region of Peru. Chemosphere.

[44] Page, C. A. Joint FAO/WHO Food Standards Programme Codex Alimentarius Commission. (2014).

[45] Rai PK, Lee SS, Zhang M, Tsang YF, Kim K-H (2019). Heavy metals in food crops: Health risks, fate, mechanisms, and management. Environ. Int..

[46] Chen H (2016). Characteristics of heavy metal transfer and their influencing factors in different soil–crop systems of the industrialization region China. Ecotoxicol. Environ. Saf..

[47] Xiao-Rui W, Sheng-Lu Z, Shao-Hua W (2016). Accumulation of heavy metals in different parts of wheat plant from the Yangtze River Delta. China. Int. J. Agric. Biol..

[48] Bermudez GM, Jasan R, Plá R, Pignata ML (2011). Heavy metal and trace element concentrations in wheat grains: assessment of potential non-carcinogenic health hazard through their consumption. J. Hazard. Mater..

[49] Aydin ME (2015). Effects of long-term irrigation with untreated municipal wastewater on soil properties and crop quality. Environ. Sci. Pollut. Res..

[50] Shi GL (2015). The transportation and accumulation of arsenic, cadmium, and phosphorus in 12 wheat cultivars and their relationships with each other. J. Hazard. Mater..

[51] Zhang Y (2018). Heavy metal accumulation and health risk assessment in soil-wheat system under different nitrogen levels. Sci. Total Environ..

[52] Lin Y-F, Aarts MG (2012). The molecular mechanism of zinc and cadmium stress response in plants. Cell. Mol. Life Sci..

[53] EPA, U. Risk assessment guidance for Superfund, vol. I: human health evaluation manual. (EPA/540/1–89/002. Office of Solid Waste and Emergency Response Avaolable at, 1989).

[54] Szkup-Jablonska M (2012). Effects of blood lead and cadmium levels on the functioning of children with behaviour disorders in the family environment. Ann. Agric. Environ. Med..

[55] Pirsaheb M, Hadei M, Sharafi K (2021). Human health risk assessment by Monte Carlo simulation method for heavy metals of commonly consumed cereals in Iran-Uncertainty and sensitivity analysis. J. Food Compos. Anal..

[56] Qin Y (2021). Metal/metalloid levels in hair of Shenzhen residents and the associated influencing factors. Ecotoxicol. Environ. Saf..

[57] Li J (2020). Urban land-use impacts on composition and spatiotemporal variations in abundance and biomass of earthworm community [J]. J. For. Res..

[58] Setia R (2021). Phytoavailability and human risk assessment of heavy metals in soils and food crops around Sutlej river. India. Chemosphere.

